# The Immediate Treatment of Sprains

**Published:** 1900-11-24

**Authors:** 


### THE IMMEDIATE TREATMENT OF SPRAINS.

Few subjects deserve more careful consideration at the hands of the practical surgeon than the treatment of sprains, especially in view of the fact, now so well established by aid of the $x$-rays, that in many cases of "sprain" a portion of the bony attachment of the ligament is torn off. Speaking recently on this subject Mr. Tubby pointed out that a sprain is not merely an injury to ligaments alone; all the structures entering into the joint, and some even outside, are apt to be affected. Wherever damage has occurred effusion is likely to take place, and thus it happens that the pain following the sprain is of two kinds: first, the immediate pain resulting from the stretching and tearing of the joints; and secondly, often after an interval of quiescence, a pain of another character, resulting from the tension and effusion that takes place. Much damage is apt to be caused during the interval of quiescence by patients attempting to "walk off" the results of the sprain.

Sprains may be treated either by aid of immobility or mobility, and Mr. Tubby is strongly in favour of the former, except in very slight cases. If a sprain be seen within two or three hours of its occurrence cold should be applied vigorously for ten minutes or so by the affusion of cold water, by the application of ice, or by the use of spirit lotion; then the joint, after being wrapped up in lint or other fabric, wet with cold water or spirit lotion, should be surrounded with three or four layers of cotton wool, and being held in that position in which the potential cavity of the joint is at the smallest, should be firmly bandaged. By this means it may fairly be hoped that the effusion will be held in check to some degree.

But when the second attack of pain—that due mainly to tension—has come on, cold will do no further good. Heat, on the other hand, will not only diminish pain, but will promote absorption. Rest and pressure should, however, be maintained. Before long, however, movement must begin, and, according to Mr. Tubby, "at an average of three or four days after the swelling has subsided movement of the joint should be commenced." We think the tendency nowadays is to commence passive movement, without pressure sooner than this. In any case, however, the point to be borne in mind is that the direction of the movement shall be such as shall not interfere with the healing of the strained and ruptured ligaments. Friction is often of service even before movement may seem proper.

